# Examining Access to Primary Care for People With Opioid Use Disorder in Ontario, Canada: A Randomized Clinical Trial

**DOI:** 10.1001/jamanetworkopen.2022.33659

**Published:** 2022-09-30

**Authors:** Sheryl Spithoff, Lana Mogic, Susan Hum, Rahim Moineddin, Christopher Meaney, Tara Kiran

**Affiliations:** 1 Department of Family and Community Medicine, Women’s College Hospital, Toronto, Ontario, Canada; 2 Department of Family and Community Medicine, University of Toronto, Toronto, Ontario, Canada; 3 Women’s College Hospital Research Institute, Women’s College Hospital, Toronto, Ontario, Canada; 4 Department of Family and Community Medicine, St Michael’s Hospital, Unity Health Toronto, Toronto, Ontario, Canada; 5 MAP Centre for Urban Health Solutions, Li Ka Shing Knowledge Institute, St Michael’s Hospital, Toronto, Ontario, Canada; 6 Institute of Health Policy, Management and Evaluation, Toronto, Ontario, Canada

## Abstract

**Importance:**

People with opioid use disorder are less likely than others to have a primary care physician.

**Objective:**

To determine if family physicians are less likely to accept people with opioid use disorder as new patients than people with diabetes.

**Design, Setting, and Participants:**

This randomized clinical trial used an audit design to survey new patient intake at randomly selected family physicians in Ontario, Canada. Eligible physicians were independent practitioners allowed to prescribe opioids who were located in an office within 50 km of a population center greater than 20 000 people. A patient actor made unannounced telephone calls to family physicians asking for a new patient appointment. The data were analyzed in September 2021.

**Intervention:**

In the first randomly assigned scenario, the patient actor played a role of patient with diabetes in treatment with an endocrinologist. In the second scenario, the patient actor played a role of a patient with opioid use disorder undergoing methadone treatment with an addiction physician.

**Main Outcomes and Measures:**

Total offers of a new patient appointment; a secondary analysis compared the proportions of patients offered an appointment stratified by gender, population, model of care, and years in practice.

**Results:**

Of a total 383 family physicians included in analysis, a greater proportion offered a new patient appointment to a patient with diabetes (21 of 185 physicians [11.4%]) than with opioid use disorder (8 of 198 physicians [4.0%]) (absolute difference, 7.4%; 95% CI, 2.0 to 12.6; *P* = .007). Physicians with more than 20 years in practice were almost 13 times less likely to offer an appointment to a patient with opioid use disorder compared with diabetes (1 of 108 physicians [0.9%] vs 10 of 84 physicians [11.9%]; absolute difference, 11.0; 95% CI, 3.8 to 18.1; *P* = .001). Women were almost 5 times less likely (3 of 111 physicians [2.7%] vs 14 of 114 physicians [12.3%]; absolute difference, 9.6%; 95% CI, 2.4 to 16.3; *P* = .007) to offer an appointment to a patient with opioid use disorder than with diabetes.

**Conclusions and Relevance:**

In this randomized clinical trial, family physicians were less likely to offer a new patient appointment to a patient with opioid use disorder compared with a patient with diabetes. Potential health system solutions to this disparity include strengthening policies for accepting new patients, improved compensation, and clinician anti-oppression training.

**Trial Registration:**

ClinicalTrials.gov Identifier: NCT05484609

## Key Points

**Question:**

Are family physicians less likely to accept people with opioid use disorder as new patients than people with diabetes?

**Findings:**

In this randomized clinical trial conducted in Ontario, Canada, assessing 383 family physicians, were almost 3 times less likely (4% vs 11%) to offer a new patient appointment to a prospective patient with opioid use disorder than those with diabetes.

**Meaning:**

These findings suggest that physician discretion in accepting new patients contributes to poor primary care access for patients with opioid use disorder, indicating a need for health system changes.

## Teaser

This randomized clinical trial uses an audit design to compare rates at which family physician offices accept new patients with opioid use disorder vs new patients with diabetes in Ontario, Canada.

## Introduction

People with opioid use disorder (OUD) are a growing population in the US and Canada, as well as other in high-income countries.^1^ A large proportion have other chronic health conditions and some receive opioid agonist treatment (OAT).^2,3,4,5,6^ Access to high-quality primary care has the potential to improve care and health outcomes for this complex population.^7,8,9,10^ A recent study, set in Ontario, Canada, found that people with OUD who were enrolled with a primary care physician were more likely to have appropriate cancer screening and diabetes monitoring than those not enrolled.^11^ Similarly, an American study found that patients with OUD were more likely to receive appropriate preventative screenings when they received integrated addiction and primary care in a federally qualified health center.^12^

Despite these findings of improved health outcomes, people with OUD have poor access to primary care. In the Canadian study cited above, 43% of people in treatment for OUD were enrolled with a family physician, compared with 73% of matched controls.^11^ Another Canadian study found that among a cohort of people who use drugs, only 56% were engaged with primary care.^13^ Similarly, access to primary care for Americans who use substances, even among insured populations, is often poor.^14,15^ Research also indicates that finding a new physician may also be difficult for those with OUD. In a 2021 cohort study, people with OUD were significantly less likely to find a new family physician in the year after their enrollment with a previous physician was terminated.^16^

Research has identified some barriers to primary care for people who use substances. Patient factors, such as difficulty attending appointments, and system barriers, like transportation costs, impede access.^17,18,19^ Health care providers’ reluctance to care for this population may also play a role. In surveys, physicians report that they do not have the skills or time to care for people with addictions or who require prescribed opioids.^20,21^ Similarly, patients who use substances or have addictions reported having encountered physicians without appropriate knowledge and being refused acceptance into a practice.^17,22,23,24^ To date, however, studies have not determined how care decisions made by primary care physicians may factor into access to care for this population.

Our study objective, therefore, was to determine if family physicians are less likely to accept people with OUD as new patients than people with diabetes, by comparing the proportion of people with OUD offered a new patient appointment vs those with diabetes. In a secondary analysis, we investigated the impact of population size, physician gender, model of care^25^ and years in practice on the likelihood of being offered a new patient appointment.

## Methods

Our study was conducted in Ontario, Canada’s largest province with a population of 15 million.^26^ Ontarians have publicly funded coverage for essential medical services including primary, emergency, and specialized care as well as for medical procedures and hospitalizations.^27^ About 91% of Ontarians report having a primary care provider, either a family physician or a nurse practitioner.^28^ Approximately 80% of Ontarians are formally enrolled to a physician practicing in an enrollment model.^29^ In enrollment models, physicians work in a group setting with shared after-hours responsibility and receive blended capitation and fee-for-service payments.^29^ About one-quarter of enrolled patients receive care in a team-based medical home (referred to as “family health teams”). Unlike other enrollment models, family health teams receive payments to hire additional staff such as nurse practitioners, counselors, and pharmacists.^25,29^

This study received ethics approval from Women’s College Hospital research ethics board, which waived informed consent by study participants. We did not register our study in advance because we did not have an experimental intervention (eg, drug, device, or behavioral intervention).

### Study Design

We conducted a randomized clinical trial using a controlled audit study design^30^ with 2 parallel arms with 1:1 allocation (Supplement 1). A patient actor made unannounced telephone calls to randomly selected family physicians across Ontario asking for a new patient appointment. For our primary analyses, we compared an offer of a new patient appointment for a patient in treatment for diabetes vs a patient in treatment for OUD. For our prespecified secondary analysis, we compared proportions of patients offered an appointment for each scenario stratified by gender, population size, model of care, and years in practice.

### Participants

Using publicly available data from the College of Physicians and Surgeons of Ontario (CPSO) website, we compiled a list of all Ontario physicians with a reported specialty in family medicine.^31^ The CPSO requires all practicing physicians in Ontario to submit updated practice information to the CPSO on a yearly basis. We also collected information on physician gender, years in practice, and practice postal code from the CPSO website. We used data from Statistics Canada on Canadian municipalities to determine the population of the physician’s primary practice setting based on the postal code. We determined whether the physician was part of a team using publicly available data from the Association of Family Health Teams in Ontario website.

### Exclusions

Using information from the CPSO website, we excluded physicians with restricted practices as many of these physicians are not allowed to prescribe opioids as well as physicians not in independent practice (ie, medical residents or trainees). We only included 1 physician per practice address. A study statistician (C.M.) grouped family physicians by primary practice address and then selected, uniformly at random (with R version 4.0.4 [R Project for Statistical Computing]), 1 family physician who worked at the practice address, excluding the others. Using data from Statistics Canada,^32^ we also excluded physicians whose practice address was in a community with a population less than 10 000, unless they were close (ie, within 50 km) to a community with a population greater than 20 000. We excluded physicians in these communities because we hypothesized that the study scenario may not be plausible given that these settings may be too small to have a methadone clinic or an endocrinologist. At the time of making the phone calls, we excluded physicians who did not provide primary care, were no longer in practice, had a voicemail stating they were not accepting new patients, required an in person visit prior to accepting a new patient, required a health card number prior to accepting new patients, or only used Health Care Connect (a government service that requires a health card number) to accept new patients (Figure). Our patient actor could not provide a health card number (a unique, government-assigned number that enables access to publicly funded health services) without compromising their privacy. We also excluded physicians if we were unable to contact them, or leave a message, after 5 phone calls.

**Figure.**
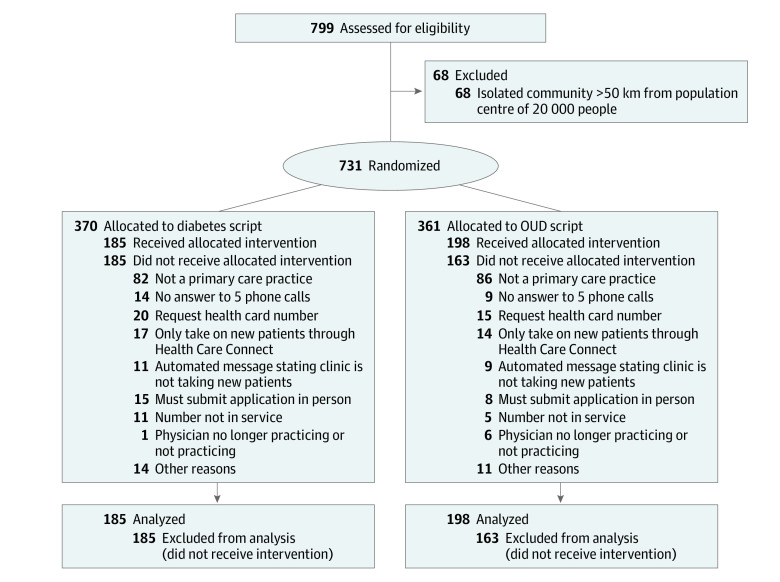
Flow Diagram of Trial Participation

### Sample Size

Our team consensus was that a 10% difference in the proportion of new patient appointments offered to a patient with diabetes vs OUD would be clinically meaningful. In a similar study design, Olah et al^30^ found that 23.5% of those with diabetes were offered an appointment with a family physician in Ontario. Thus for our sample size calculation, we assumed that 23% of those with diabetes and 13% of those in treatment for OUD would be offered a new appointment. At 80% power, 5% type-1 error level, to detect a 10% difference in the proportion of new appointments offered between opioid (13%) and diabetes (23%) groups, we estimated we would need to contact 231 physicians with each scenario. However, previous studies using this approach found that up to one-third of physicians were excluded at the time of making the phone call (eg, the physician only provided walk-in services).^30,33^ Therefore, we estimated that we needed to contact 308 physicians in each arm. However, after making initial phone calls, we revised our estimate to 400 in each arm as only about 50% of the physicians we called met our inclusion criteria.

### Sampling, Allocation, and Masking

Using the subset of eligible physicians, a study statistician (C.M.) sampled 799 family physicians, uniformly at random, and allocated them to the diabetes or OUD patient scenarios using simple randomization with a random Bernoulli sequence generator (Figure). Concealment of allocation was not possible, as the research assistant (L.M.) needed to know which scenario to implement during each phone call. The statisticians were masked to the scenario assignment when calculating the primary outcome. We made calls to the participants between June 11, 2021, and September 20, 2021. In total, 383 met our inclusion criteria and were included in the analysis. We excluded the other participants after allocation because we were unable to determine if these physicians met our inclusion criteria until after making the phone call and stating the scripted scenario (eTable 1 in Supplement 2).^30^

### Intervention Scenarios

In the first scenario, the caller followed a script where they played the role of a patient with diabetes in treatment with an endocrinologist (eTable 1 in Supplement 2). In the second scenario, the caller played the role of a patient with OUD undergoing methadone treatment with an addiction physician. One member of the research team (L.M.) made all the phone calls. We phoned a physician’s practice up to 5 times over 6 weeks. If the office had voicemail, we left a message using the sample script, but continued to call up to 5 times. We accepted call-backs up to 6 weeks after the first phone call. If a physician’s practice offered an appointment, we canceled it the next day.

### Outcomes

Our primary outcome measure was an offer of a new patient appointment with the physician we contacted, or with another physician or nurse practitioner at the same clinic. All other outcomes were considered negative including: placement on a waitlist; no call-back within 6 weeks; instructions to call back later at a date more than 6 weeks later (if less than 6 weeks, we called back); refusal to offer an appointment; suggestions to call another physician or nurse practitioner at the same clinic or another clinic; or no response to voicemail messages after six weeks. We concluded our study once we made up to 5 attempts to call these physicians.

### Statistical Analysis

We used descriptive statistics (mean for continuous measures, frequency and percentages for nominal measures) to describe the sample. To assess the balance between the 2 groups, we used χ^2^ tests and Wilcoxon tests for categorical and continuous measures, respectively. To assess if our exclusions after allocation were balanced between the 2 groups, we used χ^2^ tests for categorical measures.

For our primary outcome, we used 2-sample χ^2^ tests to compare unconditional offers of a new patient appointment for a patient in treatment for diabetes with a patient in treatment for OUD. We also used multivariable logistic regression to model the likelihood of being offered a new appointment with a physician after controlling for our prespecified confounding factors: gender, rurality, years in practice and model of care.

For our prespecified secondary analysis, we performed a stratified analysis comparing proportions of patients offered an appointment for each scenario stratified by gender (men vs women), municipality population size (rural, population below 50 000 vs urban, population above 50,000), model of care (team-based vs not), and years in practice (more than 20 years vs less than 20 years). The absolute proportion differences 95% CIs and *P* values are reported. For our analyses, we considered a *P* < .05 to be statistically significant. Statistical analyses were conducted using SAS version 9.4 (SAS Institute).

## Results

We included 383 physicians in our analysis. The reasons for exclusion after allocation were not statistically different between the 2 groups (eAppendix 2 in Supplement 2). Overall, 225 physicians (58.8%) were women, 40 (10.5%) practiced in a region with a population less than 50 000 people, and 37 (9.7%) practiced in a family health team (Table 1). Physicians had been in clinical practice for a mean (SD) average of 19.9 (10.1) years. Physician characteristics were not statistically different between the 2 groups (eAppendix 1 in Supplement 2).

**Table 1.** Characteristics of Physicians and Their Practices

Characteristics	Physicians, No. (%)		
	Overall (n = 383)	Diabetes scenario (n = 185)	OUD scenario (n = 198)
Gender			
Women	225 (58.8)	114 (61.6)	111 (56.1)
Men	158 (41.2)	71 (38.4)	87 (43.9)
Office not near population center^a^	40 (10.5)	17 (9.2)	23 (11.7)
FHT model	37 (9.7)	22 (11.9)	15 (7.6)
Time in practice, mean (SD), y	19.9 (10.1)	18.8 (10.3)	20.9 (9.9)

Abbreviations: FHT, family health team; OUD, opioid use disorder.

^a^ Population centers defined as having more than 50 000 residents.

### Primary Outcome

A greater proportion of physicians offered a new patient appointment to a caller with diabetes (21 of 185 physicians [11.4%]) than one in treatment for OUD (8 of 198 [4.0%]) (absolute difference, 7.4%; 95% CI, 2.0 to 12.6; *P* = .007) (Table 2). After controlling for gender, years in practice, practice location, and model of care, a caller presenting with diabetes had greater odds of being offered a new patient appointment (OR, 2.9; 95% CI, 1.3-6.8; *P* = .01).

**Table 2.** Stratified Secondary Analyses Rates of Offering a New Patient Appointment

Subgroup	No. of patients	Physicians, No. (%)		Absolute difference, % (95% CI)	*P* value
		Diabetes scenario (n=185)	OUD scenario (n=198)		
Total	383	21 (11.4)	8 (4.0)	7.4 (2.0 to 12.6)	.007
Gender					
Women	225	14/114 (12.3)	3/111 (2.7)	9.6 (2.8 to 16.3)	.007
Men	158	7/71 (9.9)	5/87 (5.8)	4.1 (−4.4 to 12.6)	.33
Population size					
<50 000	40	0/17	2/23 (8.7)	−8.7 (−20.2 to 2.8)	.21
>50 000	340	21/167 (12.6)	6/173 (3.5)	9.1 (3.4 to 14.8)	.002
Model of care					
Team	37	3/22 (13.6)	0/15	13.6 (−0.7 to 28.0)	.14
Not team	346	18/163 (11.0)	8/183 (4.4)	6.7 (1.0 to 12.3)	.02
Time in practice					
0-20 y	191	11/101 (10.9)	7/90 (7.8)	3.1 (−5.1 to 11.3)	.46
21-36 y	192	10/84 (11.9)	1/108 (0.9)	11.0 (3.8 to 18.1)	.001

### Stratified Analysis

In the stratified analysis, we found that women (3 of 111 physicians [2.7%] vs 14 of 114 [12.3%]; absolute difference, 9.6%; 95% CI, 2.8 to 16.3; *P* = .007) as well as physicians practicing in larger centers (6 of 173 physicians [3.5%] vs 21 of 167 physicians [12.6%]; absolute difference, 9.1; CI, 3.4 to 14.8; *P* = .002), in a non-team model (8 of 183 physicians [4.4%] vs 118 of 163 physicians [1.0%]; absolute difference, 6.7; 95% CI, 1.0 to 12.3; *P* = .02) and physicians with more years in practice (1 of 108 physicians [0.9%] vs 10 of 84 physicians [11.9%]; absolute difference, 11.0; 95% CI, 3.8 to 18.1; *P* = .001) were less likely to offer a new patient appointment to a patient with OUD compared with a patient with diabetes (Table 2). Findings were not significant for men, physicians in more rural areas, in team-based practices, and with fewer years in practice.

## Discussion

In this randomized clinical trial conducted in a setting with universal health coverage, we found that family physicians were almost 3 times less likely to offer a new patient appointment to a patient with OUD than diabetes. Our findings demonstrate that family physicians are reluctant to accept patients with OUD into their practices. Family physician discretion in accepting new patients, therefore, may be a major reason why patients with OUD take longer to find,^16^ and are less likely to have a family physician.^11^

Our study results align with findings from physician surveys. In a 2019 Canadian survey study,^34^ 28% of physicians reported they would not accept patients requiring prescribed opioids into their practice. Similarly, an American study found that over 40% of clinics would not prescribe opioids to a simulated patient seeking a new patient appointment.^35^ Physicians report concerns that patients with OUD or who require prescribed opioids may be too complex as well as disruptive to their practice.^20,21,36,37^ Physicians also report that they lack the training, skills, and support necessary to provide appropriate care to this population.^20,21^ The reluctance to accept patients with OUD into a primary care practice may also be the result of physician attitudes toward people with addictions or who are prescribed opioids.^23,38,39,40^ Health care professionals report high levels of stigma, similar to the general public, toward these populations.^20,36,41,42,43^

Compensation models in Ontario may have played a role as well. Although all Canadians have access to universal health care, studies indicate that physicians are reluctant to accept complex, high-needs patients into their practices when they do not receive appropriate compensation.^9^ In Ontario, most physicians are eligible to bill special fee codes for the care of patients with diabetes.^29,44^ Although the province also provides special fee codes for the care of people with OUD, family physicians can only bill these codes if they are the primary provider of addiction care (including OAT such as methadone), a rarity in Ontario.^45^ Physicians practicing in Ontario, therefore, may be more inclined to accept a patient with diabetes into their practice believing they will be appropriately compensated for the complexity of care.

In a secondary stratified analysis, we found that physicians who had been in practice for more than 20 years were almost 13 times less likely to offer a new patient appointment to a caller with OUD than one with diabetes. Our finding aligns with the 2019 Canadian survey study where physicians with more years in practice were less likely to accept a new patient needing treatment with opioids than physicians who had been in practice for a shorter time.^34^ These findings may indicate changing attitudes and behaviors among newly trained physicians, reflecting emerging societal norms^46,47^ or improved medical education.^48,49^ We also found that female physicians were almost 5 times less likely to offer a new patient appointment to a patient with OUD than one with diabetes. This contradicts findings in the 2019 survey study where female physicians were more likely than male physicians to report accepting patients prescribed opioids into their practices.^34^ In a UK study, gender did not appear to influence physician willingness to prescribe opioids.^50^

Our small sample size for team-based models of care found no significant difference between patients with OUD and diabetes in the stratified analysis. Other studies have found that high-needs patients are less likely to be enrolled in highly-resourced, team-based medical models.^9,16,51^ Because physicians working in these models are funded based on age and sex, and not complexity, they may be incentivized to seek out healthy patients and avoid complicated ones.^9,51^ However, in the Canadian survey described above, physicians in team-based practices were more willing than others to report accepting patients prescribed opioids into their practices.^34^ Similarly, American physicians who were part of a large health plan with substantial infrastructure supports reported being less concerned about prescribing opioids than others.^52^

Our results suggest that policymakers should implement policies ensuring that all patients within a catchment area are automatically eligible for a primary care physician.^53^ Short of this solution, policymakers and regulatory bodies should enhance policies that reduce physician discretion in accepting new patients.^51,54,55^ To ensure physicians have the appropriate resources, compensation models should better reflect patient complexity.^18,56^ Equally important, policies should include measures to ensure high-quality care for complex and stigmatized populations. These could include requirements for enhanced education in medical schools and anti-oppression training for primary care practices.^40,57,58^ Finally, researchers should assess the effectiveness of these interventions and determine if they improve access to high-quality care.

### Limitations

Our study had several limitations. First, as we were not able to determine if participants met our inclusion criteria until after making a phone call and stating the script, we excluded many participants after allocation. However, given that we did not give participants an option to opt of the study, but only excluded those who did not meet our prespecified criteria, our approach is unlikely to lead to selection bias. Furthermore, our analysis showed no statistical difference in reasons for exclusion between the two groups (eAppendix 2 in Supplement 2). Second, although physicians were our unit of analysis and are responsible for accepting new patients, it is possible that the reception staff are authorized to make these decisions. Third, we conducted our study during the COVID-19 pandemic, which may have led to low rates of new patient acceptance and contributed to nonsignificant findings for some of our secondary analyses.^30,59,60^ It is unclear how the pandemic might have affected acceptance rates of a patient with OUD vs diabetes. Finally, our findings may not be generalizable to other contexts outside of Ontario.

## Conclusions

In this study, family physicians were less likely to offer a new patient appointment to a person with OUD than with diabetes. Our findings suggest that physician discretion in accepting new patients contributes to poor primary care access for people in this marginalized population. Policymakers and professional regulatory bodies should strengthen policies for accepting new patients, enhance medical training, ensure compensation reflects patient complexity, and require clinician anti-oppression training. They should also consider removing physician discretion in accepting new patients by using a geographic catchment area model. Universal health care coverage must be combined with policies that ensure equitable access to care.

## Notes

Supplement 1.
Trial Protocol

Supplement 2. **eTable 1.** Background Information for Clinical Caller **eTable 2.** Responses to Questions **eTable 3.** Voicemail **eAppendix 1.** Physician Characteristics **eAppendix 2.** Exclusions After Allocation

Supplement 3.
Data Sharing Statement
