# Supplementary material for: Examining Access to Primary Care for People With Opioid Use Disorder in Ontario, Canada: A Randomized Clinical Trial
Source: JAMA Netw Open. 2022 Sep 30;5(9):e2233659. doi: 10.1001/jamanetworkopen.2022.33659 (PMC9526081; doi:10.1001/jamanetworkopen.2022.33659)
Supplement: Supplement 1. — Trial Protocol [file jamanetwopen-e2233659-s001.pdf]

Spithoff S, Mogic L, Hum S, Moineddin R, Meaney C, Kiran T. Examining access to primary care for people with opioid use disorder. *JAMA Netw Open.* 2022;5(9):e2233659. doi:10.1001/jamanetworkopen.2022.33659

Supplementary file Protocol

Access to primary care for people with opioid use disorder (OUD)

Study team: Sheryl Spithoff (PI), Tara Kiran (co-I), Susan Hum (co-I)

Summary

Background: Access to high quality primary care is essential for health, particularly for vulnerable populations. Research indicates, however, that people with opioid use disorder (OUD), are less likely than others to have a primary care provider. The reasons are unclear, but may be related to patient factors, system barriers and provider factors, including discrimination.
Research goal: Our primary goal is to determine if discrimination by primary care physicians plays a role in poor access to primary care for those in treatment for OUD. The answers will help researchers and policy-makers find ways to improve access to primary care for this vulnerable population.
Research question: Are people in treatment for OUD less likely to be offered a new patient appointment with a physician compared to those in treatment for diabetes?
Overall study design: In this randomized controlled trial (RCT), we will make unannounced phone calls to primary care physicians' practices to ask for a new patient appointment. Physicians will be randomly assigned to one of two clinical scenarios: a patient with diabetes, or a patient in treatment for OUD. Our outcome measure is an unconditional offer of a new patient appointment with the physician we contact or with another physician at the same practice. In an secondary analysis we will determine the impact of physician gender, years in practice, rurality and model of care on offers of a new patient appointment.
Participants: Randomly-selected primary care physicians in Ontario.
Data analysis methods: We will use chi-squared test and logistic regression to determine if there is a statistically and clinically significant difference in the proportions of offering a new patient appointment between the two clinical scenarios.

Clinical Trial Registry

As our study does not meet the World Health Organization (WHO) definition of a clinical trial, it will not be registered. However, we will create an a priori protocol and include it an appendix when we publish the study.

Study context

According to the World Health Organization (WHO) primary health care is a "highly effective and efficient way to address the main causes and risks of poor health and wellbeing today" (1). Primary care is particularly important for vulnerable populations as it appears to mitigate effects from factors like low socioeconomic status (2–4). This appears to be true for those with opioid use disorder (OUD): in previous work, we found that enrolment to a primary care physician was associated with higher rates of cancer screening and diabetes monitoring for those in treatment for OUD (5).

In universal health care systems, like Canada's, everyone is entitled to primary health care, regardless of health conditions or financial status. Our previous work, however, indicates that Ontarians in treatment for OUD are less likely to have access to primary care than others. We found that only 43% of people in treatment for OUD were enrolled with a primary care physician compared to 73% of matched controls (5). Our findings are consistent with an American study that reported poor access to primary care for those in treatment for OUD (6). These findings are concerning. Not only are those with OUD a vulnerable population with complex health needs, their numbers are also growing. OUD currently affects 15.5 million people worldwide (7), with Canada and the United States (U.S.) having the highest burden of disease (8).

Barriers to primary care for those with OUD have been poorly studied. Patient factors, such as difficulty attending appointments, and system barriers, like transportation costs, may affect access (9). Provider factors appear to play a role as well. A qualitative interview study in Ontario found that provider discrimination and stigma towards those with substance use disorder presented a barrier to accessing primary care (9). Other studies support this finding, showing that many providers, just like the general public, have stigma towards those with substance use disorder (10,11). It is possible that negative attitudes lead providers to discriminate against people with OUD when deciding whether to offer a new patient appointment.

Study rationale and goal

Our study goal is to determine if discrimination by physicians is a barrier to accessing primary care for those with OUD. If we find that provider discrimination is a barrier to care, researchers should explore reasons that lead to discrimination. These factors may include: physician funding models, education, resources, and attitudes. If discrimination at the practice level is not a barrier to care, then research and policy should focus on understanding and addressing other potential barriers, including patient and system factors.

Our proposed research, and future work based on our results, should improve access to primary care for people with OUD. For example, if we find that discrimination at the practice level is a factor, and future research demonstrates that lack of provider knowledge is a factor, policy-makers and educators may increase medical school training and academic detailing for practicing physicians. This should improve health outcomes for this growing population with complex health needs. It will also likely benefit physicians through additional resources, supports and improvements to funding models to enable them to care for this complex population. It may lead to improvements in family medicine as a whole by identifying areas for improvement in training and practice.

Research questions

1. Are people in treatment for OUD less likely to be offered a new patient appointment compared to people in treatment for diabetes?
2. What is the impact of physician gender, years in practice, rurality and model of care on offers of a new patient appointment?

Methods

We will conduct a randomized controlled trial (RCT), using a similar approach to Olah et al that looked at access to primary care for those of low socio-economic status (14). We will make unannounced phone calls to primary care physicians in Ontario asking for a new patient appointment. Physicians will be randomly assigned to one of two clinical scenarios: a patient with diabetes treated by an endocrinologist, or a patient with OUD prescribed methadone by an addiction physician. In the secondary exploratory analyses, we will also assess the impact of the model of care (team vs not), gender, rural vs urban, and years in practice.

Population

We will use information on the College of Physicians and Surgeon of Ontario (CPSO) website to identify all physicians in Ontario who have an active, independent, unrestricted practice with a self-reported speciality in family medicine (20). Using physician's primary practice address from the CPSO website, we will exclude physicians whose practice address in in a community of less than 10,000 unless within 50 km of municipality with population greater than 20,000 using the Statistics Canada website (https://www12.statcan.gc.ca/census-recensement/2016/dp-pd/hlt-fst/pd-pl/Table.cfm?Lang=Eng&T=301&S=3&O=D) because it may be more difficult to devise a plausible clinical scenario in smaller centres where there are no methadone clinics. We will exclude physicians who do not provide primary care (i.e. those who have a specialized practice that is not family medicine). We will exclude physicians whose sole practice is a walk-in clinic or who provide care to a particular group like a nursing home residents or university students.

We will also exclude physicians with restricted practices (e.g. are not allowed to accept female patients or not allowed to prescribe controlled substances). A restricted practice may affect whether or not the physician offers a new patient appointment. We will exclude physicians who require an in person visit or a health care number prior to offering a new patient appointment. We will also exclude nurse practitioners (NPs) from our sample, because information on them is not publicly available, they are likely to be influenced by different factors, since they are trained and paid differently than physicians. Additionally, they only work in some of the primary care practice models and most primary care is provided by physicians.

Sample size calculation

The objective of our study is to compare the proportion of patients offered a new patient appointment in each of the arms of the study. Our team consensus was that a 10% difference in the proportion of new patient appointments would be clinically meaningful. In a similar study design, Olah et al found that 23.5 % of those with diabetes were offered an appointment from a physician in Ontario (14). For our sample size calculation, we assumed that 23% of those with diabetes and 13% of those in treatment for OUD would be offered a new appointment. At 80% power, 5% type-1 error level, to detect 10% difference in the proportion of new appointments offered between opioid (13%) and diabetes (23%) groups, we estimated we would need to contact 231 physicians with each scenario. However, previous studies using this approach found that up to one third of physicians were excluded at the time of making the phone call (because physician worked in a walk-in clinic or had a practice that was not primary care; or because of no response after five phone calls) (14,18). Therefore, we estimate that we need to contact 308 physicians in each arm.

To create our sample, we will create a database using publicly-available information on the CPSO website. We will include all physicians who have an active, unrestricted, independent practice with speciality in Family Medicine. We will also record the physician's address(es), postal code, phone number(s), date of registration for independent practice and gender in our database. Physicians will only be identified in the database by a unique identifier. All these data items are collected by the CPSO. We will then group the physicians by primary practice address and then randomly select (using a random number generator), one physician from the list of physicians who work at the same site. We will exclude other physicians from the same practice address. We will then use a random number generator to randomly select a statistical determined sample of physicians. We will randomly assign them to one of two clinical scenarios: a patient with diabetes treated by an endocrinologist, or a patient with OUD prescribed methadone by an addiction physician.

Scenarios

We will train a research assistant (RA) in the clinical scenario and pilot-test the interaction with a simulated receptionist giving different responses (see Appendix 1: Sample Script). To reduce

confounding, we will have the same RA make all the phone calls. The RA will contact each physician to request an appointment, for one of the two clinical scenarios. We will exclude physicians at the time of making the phone call if the physician's receptionist reports that the clinic is a walk-in, or that the physician does not offer primary care (14). We will also exclude practices at the time of making the call if they request an in person visit or health card number prior to making an offer of a new patient appointment. We will attempt to reach a physician up to five times over a six-week period. For offices with voicemail, we will leave a message with the script but continue to make up to five phone calls to speak with someone in person. We will accept call-backs up to six weeks after the initial phone call. We will exclude practices where we are not able to speak to anyone or leave a voicemail after five phone calls. We will only request an appointment once from a physician. If the physician's clinic offers an appointment, we will call the next day and cancel it. We will continue sampling until we reach our target number of physicians.

Outcomes and Outcome Measures

Our outcome measure is the offer of a new patient appointment. We defined a positive outcome as an unconditional offer of a new patient appointment with a physician we contact (or with another physician or NP at the same clinic). We defined other outcomes as a negative outcome. These include: offer to place patient on a wait-list; instructions to call back later at a date more than 6 weeks after the initial phone call (if less than six weeks, we will call back); refusal to offer an appointment; and suggestions to patient to call another physician or NP at same clinic or another clinic. We will also record these outcomes.

Data analysis

We will use descriptive statistics to report on the population. To answer our research question, we will compare the proportion of patients offered a primary care appointment (a binary outcome) across the randomized groups. We will use chi-squared test and logistic regression to determine if there is a statistically and clinically significant difference in the proportions of offering a new patient appointment between the two clinical scenarios.

We expect groups to be balanced on measured and unmeasured confounding variables. Nonetheless, we will investigate the distribution of measured variables across the two groups. If we observe imbalances, we will estimate the adjusted impact on the likelihood of being offered a new appointment, after controlling for possible confounding factors.

For our secondary analyses we will use Fisher Exact test and two sample Wilcoxon test to assess if pre-specified subgroups: physician gender (male vs. female); practice location (rural < 50,000 vs. urban practice setting > 50,000); years in clinical practice, and practice model (team-based vs. not) affected the likelihood that a patient would receive an unconditional offer of a new patient appointment.

Limitations

This study will only be able to report whether or not there is a barrier to primary care at the practice level. Although physicians are the individuals who have the authority to decide whether or not to take a new patient, it is possible that the reception staff or other practice factors affect the decision. Additionally, the study will not be able to explain why there is practice-level discrimination. Reasons could include: payment model, attitudes, and access to resources. It will not reveal how that barrier developed. Further studies will be needed to answer those questions.

Milestones and Timelines

	Mar 2021	Apr 2021	May 2021	Jun 2021	Jul 2021	Aug 2021	Sep 2021	Oct 2021	Nov 2021
Obtain REB approval	■	■	■						
Collect data from website		■	■						
Hire RA				■					
Create collection form				■					
Pilot-test scenarios				■					
Collect and enter data				■	■				
Analyze data						■			
Create poster						■			
Write-up for publication							■	■	■
Create briefing report									■

REB = research ethics board, CPSO= College of Physicians and Surgeons of Ontario, RA = research assistant

Ethical Considerations

We will submit our study to the Research Ethics Board (REB) at Women's College Hospital in Toronto, Ontario for ethics approval. The major ethical consideration is the use of deception to request a new patient appointment. However, the deception is essential to our study: if we simply present the providers with the two clinical scenarios, the responses would not reflect what happens in actual practice, as people act differently when they are being observed (14). And more importantly, our study meets all the criteria set out by the Tri-Council Policy Statement (TCPS 2 (2018)) for alterations to requirements for consent. The risk to participants is minimal; the alteration to consent is unlikely to cause harm; it is impossible to conduct the research if prior consent is required; the nature of the alteration is clearly defined; and there is a plan to provide a de-briefing letter. Additionally, this type of deception has received ethical approval in the past (14,18).

In our study, participants will receive a de-briefing letter describing the study, but they will not have the option to withdraw their data. Allowing sites to remove data could inadvertently skew the data. Additionally, as the practice site is the unit of analysis, and not an individual, it is unclear who would be able to make the decision to remove data (see Appendix 2: De-brief). We will provide a contact for Women's College Hospital's REB, as well as the study PI and the study coordinator. We will offer to send the practice sites a copy of our study findings.
