# Supplementary material for: Examining Access to Primary Care for People With Opioid Use Disorder in Ontario, Canada: A Randomized Clinical Trial
Source: JAMA Netw Open. 2022 Sep 30;5(9):e2233659. doi: 10.1001/jamanetworkopen.2022.33659 (PMC9526081; doi:10.1001/jamanetworkopen.2022.33659)
Supplement: Supplement 2. — eTable 1. Background Information for Clinical Caller eTable 2. Responses to Questions eTable 3. Voicemail eAppendix 1. Physician Characteristics eAppendix 2. Exclusions After Allocation [file jamanetwopen-e2233659-s002.pdf]

Supplemental Online Content

Spithoff S, Mogic L, Hum S, Moineddin R, Meaney C, Kiran T. Examining access to primary care for people with opioid use disorder in Ontario, Canada: a randomized clinical trial. *JAMA Netw Open.* 2022;5(9):e2233659.
doi:10.1001/jamanetworkopen.2022.33659

eTable 1. Background information for clinical caller

eTable 2. Responses to questions

eTable 3. Voicemail

eAppendix 1. Physician characteristics

eAppendix 2. Exclusions after allocation

This supplemental material has been provided by the authors to give readers additional information about their work.

© 2022 Spithoff S et al. *JAMA Network Open.*

eTable 1. Background information for clinical caller

	Clinical scenario one	Clinical scenario two
**Demographics**	25 year-old woman<br>DOB: October 1st 1995<br>Phone: research cell #	25 year-old woman<br>DOB: October 1st 1995<br>Phone: research cell #
**Personal history**	Single, no children<br>Employed as cashier at grocery store<br>Some college education at Seneca<br>Parents live in Hamilton	Single, no children<br>Employed as cashier at grocery store<br>Some college education at Seneca<br>Parents live in Hamilton
**Medical history**	Diagnosed with addiction to Oxycontin age 20, treated with methadone by addiction specialist	Diagnosed with type one diabetes age 12, treated with insulin by endocrinologist
**Medications**	Methadone	Insulin
**Surgical history**	Appendectomy age 22	Appendectomy age 22
**Substance history**	Does not smoke, rarely drinks alcohol, no other substances	Does not smoke, rarely drinks alcohol, no other substances

© 2022 Spithoff S et al. *JAMA Network Open.*

eTable 2. Responses to questions

Questions from physician's office	Clinical caller response
Hello this is (CLINIC NAME) medical clinic	Hi, I am moving to (CITY NAME) in 3 weeks and my methadone doctor says I should get a family doctor.<br><br>Hi, I am moving to (CITY NAME) in 3 weeks my diabetes specialist says I should get a family doctor.
What is your name?	Lana Smith
How old are you?	25
What is your date of birth	October 1st 1995
Who was your previous family doctor?	I don't have one. I was going to walk-in clinics
Do you live in the area?	I am moving to the area in 3 weeks
Where are you moving from?	Toronto [if location outside of Toronto]<br>Hamilton [if location in GTA]
When will you move?	Three weeks
When are you available to come in?	Anytime
Can you come in today and see the doctor?	I can't come in today. Do you have an appointment for any other time?
Can I get back to you?	Yes, please call me on my cell number
Where can I reach you?	(416) 275-8753
Have you tried another family doctor?	Not yet
Do you have a health card?	Yes
Do you have any other health problems?	No, just diabetes—but I have a specialist who treats me for that<br><br>No, just methadone for my past oxy use—but I have a specialist who treats me for that
What kind of diabetes do you have?	Type one diabetes. I take insulin.
Who looks after your diabetes?<br><br><br>Who looks after your methadone?	A specialist in [Toronto/Hamilton]. I have video appointments with her.<br><br>A specialist in [Toronto/Hamilton]. I have video appointments with her.<br>[monthly appointment if they ask you]
What's your specialist's name?	Dr. Robertson
What's her first name?	Not sure. Lisa maybe?
Do you have to give urine drug tests? [for methadone scenario]	Yes, every two months at a lab
Do you take any medications?	Insulin only

© 2022 Spithoff S et al. *JAMA Network Open.*

	Methadone only
Are you looking for the family doctor to prescribe you insulin?<br><br>Are you looking for the family doctor to prescribe you methadone?	No, my specialist will do that.<br><br><br>No, my specialist will do that.
Will you find a specialist in this area?	Maybe, but for right now my specialist will keep treating me over video.
Yes, Dr. X is taking new patients. We can schedule you for (date/time). Will that work for you?	Yes, thank you
Sorry, we are not taking new patients	Ok, thank you
Ok, let me get your information for our wait-list	Ok, thank you
Please call back date/time	Ok, thank you
Please call clinic (CLINIC NAME), they might be taking new patients	Ok, thank you
Sorry this is Dr X's administrative office, please call Dr X's clinic.	Do you have the number for the clinic?<br>Do you know the name of the clinic?
This clinic charges a fee to register (eg Cleveland clinics)	Ok, thank you, I will try somewhere else then
I am going to transfer you to Dr [X] who has some questions for you	Ok, thank you

© 2022 Spithoff S et al. *JAMA Network Open.*

eTable 3. Voicemail

Voicemail	Response
Please leave a message after the beep	Hi, my name is Lana Smith<br><br>I am moving to (CITY NAME) in 3 weeks and my methadone doctor says I should get a family doctor.<br>OR<br>Hi, I am moving to (CITY NAME) in 3 weeks my diabetes specialist says I should get a family doctor.<br><br>My number is [research cell number]<br><br>Thank you.

© 2022 Spithoff S et al. *JAMA Network Open.*

eAppendix 1. Physician characteristics

Table of Scenario by Gender

Frequency<br>Percent<br>Row Pct<br>Col Pct	Scenario(Scenario)	Gender(Gender)		
		**Female**	**Male**	**Total**
	**0**	114	71	185
		29.77	18.54	48.30
		61.62	38.38	
		50.67	44.94	
	**1**	111	87	198
		28.98	22.72	51.70
		56.06	43.94	
		49.33	55.06	
	**Total**	225	158	383
		58.75	41.25	100.00

Statistics for Table of Scenario by Gender

Statistic	DF	Value	Prob
**Chi-Square**	1	1.2204	0.2693

Table of Scenario by Population__50_000__yes___no_

Frequency<br>Percent<br>Row Pct<br>Col Pct	Scenario(Scenario)	Population__50_000__yes___no_(Population >50,000 (yes / no))		
		**No**	**Yes**	**Total**
	**0**	17	167	184
		4.47	43.95	48.42
		9.24	90.76	
		42.50	49.12	
	**1**	23	173	196
		6.05	45.53	51.58

© 2022 Spithoff S et al. *JAMA Network Open.*

	11.73	88.27	
	57.50	50.88	
**Total**	40	340	380
	10.53	89.47	100.00

Frequency Missing = 3

Statistics for Table of Scenario by Population__50_000__yes___no_

Statistic	DF	Value	Prob
**Chi-Square**	1	0.6276	0.4283

Table of Scenario by Family_Health_Team

Frequency / Percent / Row Pct / Col Pct

Scenario(Scenario)	Family_Health_Team(Family Health Team)		
	**No**	**Yes**	**Total**
**0**	163	22	185
	42.56	5.74	48.30
	88.11	11.89	
	47.11	59.46	
**1**	183	15	198
	47.78	3.92	51.70
	92.42	7.58	
	52.89	40.54	
**Total**	346	37	383
	90.34	9.66	100.00

Statistics for Table of Scenario by Family_Health_Team

Statistic	DF	Value	Prob
**Chi-Square**	1	2.0415	0.1531

TWO SAMPLE WILCXON TEST IS USED TO COMPARE THE YEARS IN PRACTICE:

© 2022 Spithoff S et al. *JAMA Network Open.*

Wilcoxon Two-Sample Test					
Statistic	Z	Pr < Z	Pr > |Z|	t Approximation	
				Pr < Z	Pr > |Z|
33413.00	-1.9469	0.0258	0.0515	0.0261	0.0523

THIS IS PVALUEZ includes a continuity correction of 0.5.

Kruskal-Wallis Test		
Chi-Square	DF	Pr > ChiSq
3.7922	1	0.0515

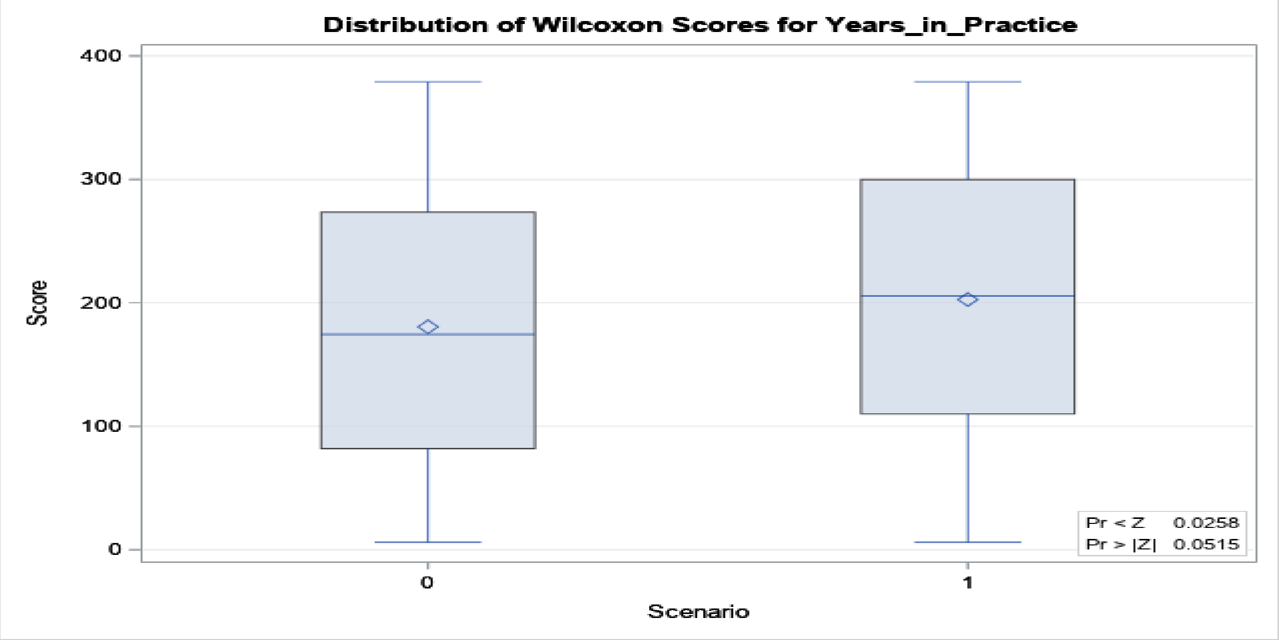

Overall there is no difference in participant characteristics between the two scenarios.

© 2022 Spithoff S et al. *JAMA Network Open.*

eAppendix 2. Exclusions after allocation

Table of reason by group

**Frequency<br>Percent<br>Row Pct<br>Col Pct**	**reason**	**group: Diabetes**	**OUD**	**Total**
	**No answer to five phone calls**	14<br>4.02<br>60.87<br>7.57	9<br>2.59<br>39.13<br>5.52	23<br>6.61
	**Not a primary care practice**	82<br>23.56<br>48.81<br>44.32	86<br>24.71<br>51.19<br>52.76	168<br>48.28
	**Only take on new patients thr**	17<br>4.89<br>54.84<br>9.19	14<br>4.02<br>45.16<br>8.59	31<br>8.91
	**Other reasons**	52<br>14.94<br>57.14<br>28.11	39<br>11.21<br>42.86<br>23.93	91<br>26.15
	**Request health card number**	20<br>5.75<br>57.14<br>10.81	15<br>4.31<br>42.86<br>9.20	35<br>10.06
	**Total**	185<br>53.16	163<br>46.84	348<br>100.00

Statistics for Table of reason by group

**Statistic**	**DF**	**Value**	**Prob**
**Chi-Square**	4	2.6638	0.6156
**Likelihood Ratio Chi-Square**	4	2.6702	0.6144
**Mantel-Haenszel Chi-Square**	1	0.8585	0.3541
**Phi Coefficient**		0.0875	
**Contingency Coefficient**		0.0872	

© 2022 Spithoff S et al. *JAMA Network Open.*

Statistic	DF	Value	Prob
**Cramer's V**		0.0875	

Sample Size = 348

Pvalue is 0.6156

© 2022 Spithoff S et al. *JAMA Network Open.*
