# Supplementary material for: Examining Access to Primary Care for People With Opioid Use Disorder in Ontario, Canada: A Randomized Clinical Trial
Source: JAMA Netw Open. 2022 Sep 30;5(9):e2233659. doi: 10.1001/jamanetworkopen.2022.33659 (PMC9526081; doi:10.1001/jamanetworkopen.2022.33659)
Supplement: Supplement 3. — Data Sharing Statement [file jamanetwopen-e2233659-s003.pdf]

Spithoff S, Mogic L, Hum S, Moineddin R, Meaney C, Kiran T. Examining access to primary care for people with opioid use disorder. *JAMA Netw Open.* 2022;5(9):e2233659. doi:10.1001/jamanetworkopen.2022.33659

DATA SHARING STATEMENT

Upon request, we will make available a limited dataset with participant identification numbers, outcomes and exclusions. We will also supply our statistical analyses.
